# Raman microspectroscopy for species identification and mapping within bacterial biofilms

**DOI:** 10.1186/2191-0855-2-35

**Published:** 2012-07-13

**Authors:** Brooke D Beier, Robert G Quivey, Andrew J Berger

**Affiliations:** 1 The Institute of Optics, University of Rochester, 275 Hutchison Rd., Rochester, NY 14627, USA; 2 Center for Oral Biology, University of Rochester Medical Center, 601 Elmwood Ave., Rochester, NY 14642, USA

**Keywords:** Raman spectroscopy, Confocal microscopy, Biofilms, Bacteria, Dental plaque

## Abstract

A new method of mapping multiple species of oral bacteria in intact biofilms has been developed, using the optical technique of confocal Raman microscopy. A species classification algorithm, developed on dried biofilms, was used to analyze spectra of hydrated biofilms containing two microbial species central to dental health: *Streptococcus sanguinis* and *Streptococcus mutans*. The algorithm transferred successfully to the hydrated environment, correctly identifying the species of origin of single-species biofilms. We then used the algorithm successfully both to detect the presence of two species in mixed biofilms and to create spatial maps within these biofilms.

## Introduction

The identification of microbial species within sample specimens is relevant to both the microbiological research laboratory and the clinical setting. A number of standard methods of species identification are currently used. Selective plating may be used to identify the constituent species and to obtain an order-of-magnitude estimate of the initial concentrations of bacteria present in a sample. Since this method involves serial dilutions, it can take up to several days and is insensitive to cells that are dead or incapable of reproduction by the time the sample is procured. Quantitative polymerase chain reaction (qPCR) is highly sensitive to the initial concentrations of known species, but it requires prior knowledge of their genomes in order to provide specificity. Both qPCR and selective plating disrupt the initial sample’s architecture, and are thus ill-suited for spatial mapping studies of specimens such as biofilms. Fluorescence *in situ* hybridization (FISH) can provide spatial resolution, but requires sample fixation (eliminating the potential to study a sample over time), extensive preparation steps, and a genetically-targeted exogenous marker. A new measurement method that could map the spatial distribution of multiple species in intact, unfixed specimens in a label-free, non-contact manner would therefore be valuable. Such a technique could also make longitudinal study of samples like biofilms a possibility.

Optical spectroscopy offers the ability to acquire chemically-specific information at micron-scale resolution without sample contact. One such method is Raman spectroscopy, which has been used extensively for biological applications. Raman spectroscopy detects the presence of molecular bonds via the inelastic scattering of laser light; each molecule imparts a different “fingerprint pattern” on the spectrum of the scattered light. This provides specificity to subtle biochemical differences between samples, useful in discrimination of cell types or monitoring the progression of disease. Raman spectroscopy has been used to study biomedical specimens including tissues, biofluids, and bacterial cells (Maquelin et al. 2002; Rösch et al. 2005; Willemse-Erix et al. 2009); extensive reviews are provided by Hanlon et al. ( 2000) and by Ellis and Goodacre ( 2006).

In the work described here, Raman spectroscopy implemented through a confocal microscope has been used to distinguish between two species of streptococci, *Streptococcus sanguinis* and *S. mutans*, grown in biofilm form. These bacteria are of particular interest due to their relationship with oral health, being the two most populous species present within dental plaque (Socransky and Manganiello 1971). *Streptococcus mutans* has been identified as the most cariogenic species in plaque, with elevated levels being linked to increased risk of tooth decay (Loesche 1986; Marsh 1999). *Streptococcus sanguinis* is associated with being the primary component of healthy plaque (Socransky and Manganiello 1971). Examining the relative balance between these two species can provide an indication of a patient’s risk of tooth decay.

Previous work by our group led to the successful identification of the species of bacteria present in single-species biofilms that had been dehydrated before analysis (Beier et al. 2010). In that study, biofilm samples had been transferred from their original substrates, much like a plaque scraping might be harvested from a tooth surface. Dehydrated samples were used for training the species identification model because sample volumes would contain denser cellular content leading to higher signal levels. Since only single-species biofilms were used in the construction of the training set, we chose this enhancement in signal levels over the preservation of structural information. Here, we apply this model to map species distributions within simple hydrated biofilms. Confirmation of the model’s successful transfer is performed using intact single-species biofilms. With the ability to identify the species within hydrated biofilms *in situ*, the model is used to perform species identifications of volumes within a two-species mixed biofilm.

## Materials and methods

### Biofilm preparations

*Streptococcus mutans* ATCC strain UA159 and *S. sanguinis* ATCC strain 10904 were examined in these studies. Although the two species have different preferred growth media, identical preparations were used when preparing pure biofilms of each species. The same nutrients were also used when creating two-species biofilms. This was done to ensure that the chemical differences detected by Raman spectroscopy were in fact indicators of differences in the species’ biofilms and not an artifact from differences in the chemical content of the nutrients used.

#### Single-species biofilms

Cells were taken from stocks stored at -80°C and streaked onto agar plates containing brain heart infusion medium (BD Difco, Franklin Lakes, New Jersey). After 24 hr, a few colonies were selected and used to inoculate a liquid culture containing 10 mL of Todd Hewitt (TH) broth (VWR International, West Chester, Pennsylvania) with 0.5% (w/v) sucrose. The presence of sucrose allows the bacteria in suspension to begin secretion of extracellular polysaccharides (EPS) that are vital for the formation of a biofilm structure. After another 24 hr, 1 mL of the resuspended liquid culture was added to 49 mL of TH broth with 0.5% sucrose. At this point, a microscope slide was introduced into the culture to serve as a substrate for the biofilm. The biofilm slide was moved to fresh media every 24 hr until a total of 4 days of growth on the substrate had been reached. In the calibration set used previously to develop our species prediction model, the sugar had been changed for the last three days to 0.5% glucose to produce more cell-rich biofilms with suppressed levels of EPS. For the sample set consisting of intact biofilms presented here, however, the sugar source was maintained as 0.5% sucrose throughout to encourage the biofilms to be more robust and space-filling, as we could no longer rely upon dehydration-induced sample compaction to increase the sample density and thus the Raman signal strength. The difference in EPS levels between the two data sets was thus a potential problem for successful calibration transfer.

For the biofilms in the calibration set, ordinary glass microscope slides were used since each biofilm was ultimately transferred from the substrate before Raman analysis, much like a plaque scraping might be transferred. For the hydrated biofilms of interest in these studies (examined *in situ*), quartz microscope slides were chosen in order to avoid the high levels of fluorescence associated with using NIR illumination on a glass substrate.

#### Two-species biofilms

Previous work by our group included the creation of artificially mixed, two-species biofilms where pure biofilms of each species were manually combined in a controlled way immediately before study, in order to investigate the spatial capabilities of our system. In that study, correct identifications of species were reliably made for locations as close as 2*μ*m to a boundary between species (Beier et al. 2010). It is of course more biologically relevant to consider multispecies biofilms grown from a common culture. In the studies described here, two-species biofilms of *S. sanguinis* and *S. mutans* were grown by first initiating a biofilm of *S. sanguinis* for 3 days before introducing liquid culture of *S. mutans*. This time delay was necessary because for the batch growth conditions used throughout these studies, *S. mutans* would quickly dominate over *S. sanguinis* due to *S. mutans* ’s proclivity for thriving in acidic environments. It was found that the delayed introduction of *S. mutans* followed by 12 hr of coexistence led to a biofilm that contained sufficient amounts of both species. Similar sample preparations consisting of 4 days of *S. sanguinis* and 3 hr coexistence with *S. mutans* or 2 days of *S. sanguinis* and 3 days of coexistence with *S. mutans* led to biofilms that were indistinguishable from pure biofilms of *S. sanguinis* and *S. mutans*, respectively.

### System design

**Figure 1:**
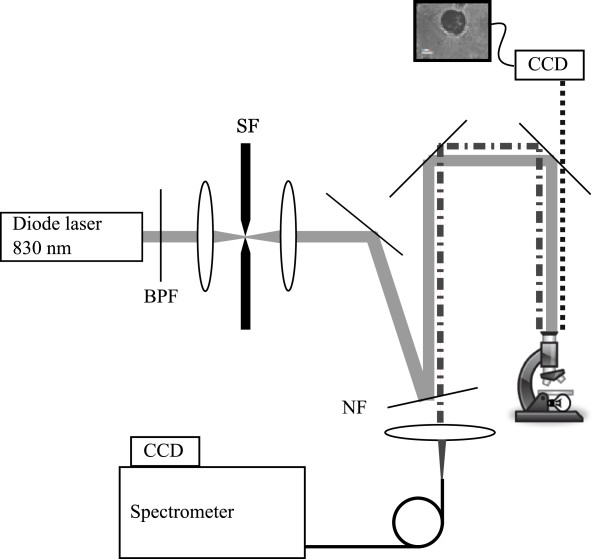
**Confocal Raman microscope.** Schematic overview of confocal Raman microscope; see text for details. Abbreviations: BPF, bandpass filter; SF, spatial filter; NF, notch filter.

Raman spectroscopy was performed using a homebuilt confocal Raman microspectroscopy system described previously (Beier and Berger 2009) and shown schematically in Figure 1. An 830 nm diode laser (Innovative Photonic Solutions, Monmouth Junction, New Jersey) was used as the excitation source for Raman scat- tering. A near-IR wavelength was chosen in order to prevent thermal effects on samples studied *in situ*. This wavelength also offered the advantage of avoiding fluorescence that is commonly observed in biological materials. The laser was directed through a spectral bandpass filter (Chroma Technology Corp., Bellows Falls, Vermont) and a spatial filter (10x objective, Newport Corp., Irvine, California; 10*μ*m pinhole). The beam was reflected from a notch filter (Semrock, Inc., Rochester, New York) at near-normal incidence before being directed into the upright microscope (Eclipse E400, Nikon Instruments Inc., Melville, New York). The beam was then focused by a 60x, 1.0 numerical aperture (NA) water immersion objective (Nikon Corp., Tokyo, Japan). The focal spot at the sample plane was ∼1.5*μ*m in diameter, delivering ∼40 mW of laser power to the sample. Epidirected Raman scattered light (Stokes-shifted in wavelength) was then collected by the same objective and directed to pass through the notch filter before being focused onto the 100*μ*m core of a multimode optical fiber, which served as a confocal pinhole. The fiber guided the light to a spectrometer (HoloSpec f/1.8, Kaiser Optical Systems Inc., Ann Arbor, Michigan) connected to a thermoelectrically cooled, front-illuminated, open electrode charge-coupled device (CCD) array (DU420-OE, Andor Technology PLC, Belfast, Northern Ireland) that was used to record the spectra. The CCD and stage were controlled using code written in-house within MATLAB Ⓡ(Version 7.8.0, The MathWorks TM, Inc., Natick, Massachusetts).

The system has a spectral resolution of ∼7 cm^−1^, as measured from neon gas emission lines. The axial sectioning depth is ∼7*μ*m, as determined from the derivative of the response curve when scanning into plastic, following the method described by Caspers et al. ( 2000). Combining the axial sectioning depth with the focal spot diameter of ∼1.5*μ*m gives a confocal volume, or voxel size, of ∼8*μ*m^3^ to be probed in each location. Scanning of the samples was achieved through the use of an electronically-controlled stage (x-y: Applied Scientific Instrumentation, Eugene, Oregon; z: Nikon).

Microscope slides fully coated by biofilm material were loaded with enough water to maintain water-immersion at the microscope objective for many hours. Sufficient air space was provided underneath the slide to drain away water seepage and prevent motion artifacts. *In situ* study of these samples meant that the structure of the biofilms, including species distributions in two-species biofilms, was intact and available for study. Scanning was performed to create maps of slices perpendicular to the substrate (XZ scans) since the structures in this orientation would be more biologically interesting, in terms of the arrangement of bacteria in relation to the substrate.

### Data acquisition and processing

Spectra were acquired for six frames of 30 s per voxel (i.e. confocal volume). The spectra were then subjected to preprocessing including cosmic-ray and system background removal, as well as spectral throughput correction. Fluorescent background was removed using a modified polynomial-fitting method described previously (Beier and Berger 2009), making use of the photobleaching lineshape between successive frames as a background fitting parameter. After the frames were preprocessed individually, they were averaged to give one spectrum per voxel. Due to a slight shift in the laser’s excitation wavelength from that used for the training set, all spectra were then recalibrated to align the 1003 cm^−1^ phenylalanine peak (Wagner et al. 2009) and resampled to a common wavenumber axis. For further analysis, only data from the wavenumber region of 706 to 1810 cm^−1^was retained for each spectrum.

Some voxels were found to have insufficient signal levels for reliable species prediction, likely due to low cellular content in a given voxel or insufficient light penetration into deeper regions. An initial screening step was performed to reject such voxels from further consideration.

After the spectral data was preprocessed, it was submitted to the species identification model described previously (Beier et al. 2010). Briefly, principal component (PC) analysis was used for data compression and noise reduction before submitting the PC scores to logistic regression for species prediction.

## Results

**Figure 2:**
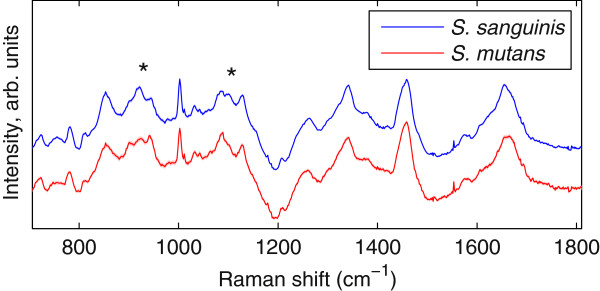
**Mean Raman spectra.** Mean Raman spectra for *S. sanguinis* and *S. mutans* from the validation set of hydrated single-species biofilms. While certain spectral regions are visibly different (marked with ‘*’), multivariate methods using the full spectra were used for species classification.

As mentioned above, the species prediction model was originally constructed from dehydrated biofilm samples and was here applied to the study of hydrated biofilms, *in situ*. For the transfer of this model to a new sample preparation, a test set of single-species biofilms was first examined. The mean spectra of the newly-measured *S. sanguinis* and *S. mutans* biofilms, shown in Figure 2, appear nearly identical. In such cases, ad hoc classification models based upon a few visibly different peaks (e.g. near 920 and 1100 cm^−1^, marked with ‘*’) perform poorly at the single-spectrum level, where noise is higher. Figure 2 emphasizes the importance of using a multivariate model (the PC-based technique referenced in the previous section) to utilize information throughout the full spectrum in constructing the classification formula.

Table 1 shows the species identification results obtained for the new test set of 352 spectra from regions within 10 different single-species biofilms. The diagonal entries in this confusion matrix represent Raman-based species assignments that agree with the (known) species from which the biofilm was prepared, while off-diagonals represent errors. 92% of all voxels of *S. sanguinis* and 94% of *S. mutans* were properly identified based on their Raman spectra. Considering all voxels from either species, 93% were properly identified. This study therefore established the baseline error rate in classification to be around 7%.

**Table 1:** Species identification performance

	**Known species**	
	***S. sanguinis***	***S. mutans***
Predicted		
*S. sanguinis*	236	6
*S. mutans*	20	90

Confusion matrix summarizing the performance of the species identification model on hydrated, intact biofilms. The columns indicate the known/true species, while the rows indicate the prevalence of experimentally predicted species in reference to the known species.

**Figure 3:**
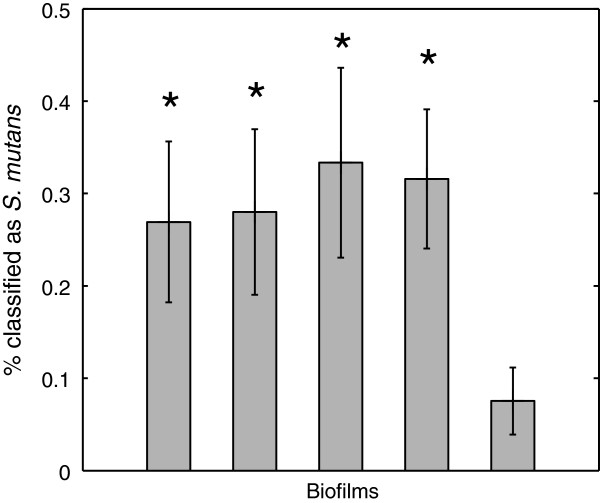
**Species predictions.** Species prediction results for 5 two-species mixed biofilms. A normal approximation to the binomial distribution has been used to determine the standard error in the mean. Biofilms with proportions of *S. mutans* statistically significantly different from results seen in pure *S. sanguinis* biofilms are marked by ‘*’.

**Figure 4:**
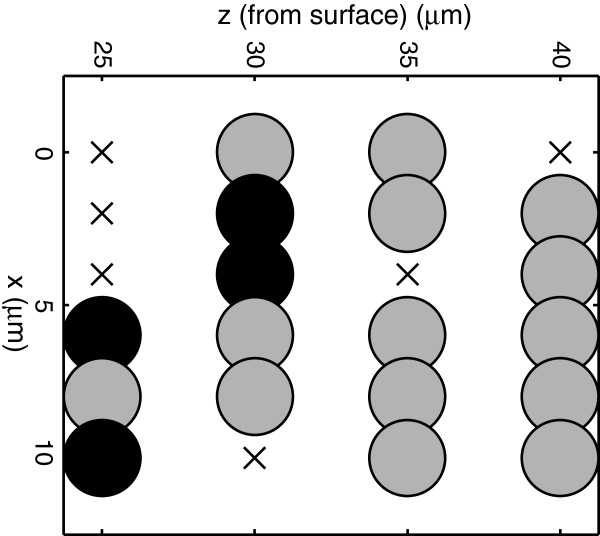
**Scan through a mixed biofilm.** XZ scan through a mixed biofilm. Gray: *S. sanguinis*; black: *S. mutans*; ‘x’: insufficient signal. Although *S. mutans* was introduced later, locations classified as *S. mutans* were closer to the substrate.

After confirming the successful transfer of the original model to working with hydrated samples, further studies examined biofilms from mixed culture. A total of five mixed biofilms were studied, each at a number of positions both laterally and in depth. Figure 3 plots the percentage of voxels that were classified as *S. mutans* in each biofilm. As the figure shows, four of the five mixed-species biofilms had Raman-assigned minority-species levels that significantly exceeded the 7% baseline error rate (Student’s t-test, 95% confidence). The spatial organization of species assignments was also examined. Figure 4 plots the Raman-based species assignments in a depth-slice through one of the mixed-species biofilms. As can be seen, the species assignments tended to be clustered into contiguous spatial regions as opposed to being evenly distributed. Such assignments are plausible, given the nature of cell proliferation, although there is no reference method currently available to provide confirmation.

## Discussion

Examination of the test set of hydrated biofilm samples showed that overall, 93% of voxels were properly identified using the transferred model. Our previous work saw 96% correct identification of species (Beier et al. 2010). The move to the new sample type involved several changes relative to the original training set, including a replacement of the laser with a corresponding 2 nm wavelength difference in excitation, the use of a water-immersion objective rather than air-immersion, the addition of depth scanning, and a decrease in signal level associated with decreased concentrations within hydrated biofilms. The high performance on this single species confirmation set given these system changes indicates the robustness of the prediction model.

With the ability to properly identify the species within hydrated samples thus confirmed, biofilms from a mixed culture were analyzed. As mentioned above, the spatial arrangement of clusters of like-classification was a reasonable result, though it was not directly verifiable. What can be asserted with confidence, however, is that in mixed-species biofilms the “minority” species was typically assigned frequently enough to exceed the baseline error rate of 7% seen in single-species studies. The fact that four of the five mixed-species biofilms had Raman-assigned minority-species levels that significantly exceeded 7% (Figure 3) indicates that the presence of both species has been detected and quantified in the mixed biofilm samples. The fifth biofilm, which did not see a statistically significant level of the minority species, may simply have been scanned in a region occupied by a single species.

There is another interesting element to the depth scan in Figure 4. Although *S. mutans* was introduced days after *S. sanguinis*, the locations classified as *S. mutans* tended to be closer to the substrate, as if the *S. mutans* had migrated beneath the layer of *S. sanguinis*. This observation was consistent across all four of the biofilms in which significant levels of both species were observed. For the particular species in this study, this could potentially have significance as it relates to the cariogenic properties of dental plaque. Further experiments are needed to explore this initial observation.

In summary, Raman spectroscopy has been implemented through a confocal microscope and used to successfully classify oral bacteria in hydrated biofilms of one or two species. A model constructed with spectra from dehydrated single-species biofilms has been transferred for the species prediction of hydrated samples. In a test set of single-species hydrated biofilms, voxel-by-voxel species assignments were 93% accurate.

When two-species biofilms from mixed culture were examined, the presences of both *S. sanguinis* and *S. mutans* were detected in four out of five biofilm regions. We cannot rule out the possibility that the remaining biofilm was simply scanned over a single-species region. The spatial arrangement of species observed in these mixed biofilms has potential implications for the study of dental plaque cariogenicity, though this aspect of our study would require further investigation. To the best of our knowledge, this is the first time two bacterial species of the same genus and sub-genus group have been mapped in a biofilm using Raman spectroscopy.

While the specific experiments described above have shown the discrimination between *S. sanguinis* and *S. mutans*, the method presented here could be applied to the study of other microbes. Although the formation of Raman-based species maps is not fast, the technique offers the ability to use intrinsic chemical differences between cells to create multidimensional maps of microbial structures without extensive knowledge of the cells’ genomes and without requiring any invasive sample preparation that could potentially alter the sample under study.

## Competing interests

The authors declare that they have no competing interests.
